# Atomic Details of Carbon-Based Nanomolecules Interacting with Proteins

**DOI:** 10.3390/molecules25153555

**Published:** 2020-08-04

**Authors:** Luigi Di Costanzo, Silvano Geremia

**Affiliations:** 1 Department of Agricultural Sciences, University of Naples Federico II, 100, 80055 Portici, Italy; 2 Centre of Excellence in Biocrystallography, Department of Chemical and Pharmaceutical Sciences, University of Trieste, 34127 Trieste, Italy; sgeremia@units.it

**Keywords:** ^129^Xe-cryptophane, calixarene, cyclodextrin, cucurbituril, fullerene, macromolecules, molecular tweezer, nanomolecules, protein crystallography, porous structure

## Abstract

Since the discovery of fullerene, carbon-based nanomolecules sparked a wealth of research across biological, medical and material sciences. Understanding the interactions of these materials with biological samples at the atomic level is crucial for improving the applications of nanomolecules and address safety aspects concerning their use in medicine. Protein crystallography provides the interface view between proteins and carbon-based nanomolecules. We review forefront structural studies of nanomolecules interacting with proteins and the mechanism underlying these interactions. We provide a systematic analysis of approaches used to select proteins interacting with carbon-based nanomolecules explored from the worldwide Protein Data Bank (wwPDB) and scientific literature. The analysis of van der Waals interactions from available data provides important aspects of interactions between proteins and nanomolecules with implications on functional consequences. Carbon-based nanomolecules modulate protein surface electrostatic and, by forming ordered clusters, could modify protein quaternary structures. Lessons learned from structural studies are exemplary and will guide new projects for bioimaging tools, tuning of intrinsically disordered proteins, and design assembly of precise hybrid materials.

## 1. Introduction

Since the discovery of buckminsterfullerene in 1985, a discrete molecule made of 60 atoms of carbon arranged to form a *I*_h_ symmetrical hollow sphere with surprising properties, it has become a favorite subject in nanotechnology and related disciplines [1]. The truncated icosahedral fullerene sphere has a van der Waals diameter of about one nanometer and several variants of smaller and larger diameters are known including elongated-shaped molecules recognized as nanotubes [2,3]. The chemical structure of these carbon-only molecules make them better conductors of electricity than common metals on much smaller scale [4]. The interest for these nanomolecules is consequence of their light weights, making them ideal for technological applications as well as biology and related fields applications [5,6]. By the 2000s, a number of studies had explored chemical strategies to link proteins and nucleic acids to nanomolecules, including metal clusters, with the aim to engineer devices for medical and biotechnological applications [7].

Among emerging carbon-made nanostructures, graphene, a single carbon sheet derived from graphite, and its related graphene oxide are very promising materials for tissue engineering, drug delivery, nerve tissue regeneration and biosensing [8,9,10,11].

Major obstacles with use of carbon-only nanomolecules for biological and medical purposes include their very poor solubility in water and poor affinity for a given protein target.

The coupling of fullerene with chemical polar groups or using water soluble capsules hosting fullerene facilitated preparation of water fullerene solutions [12,13,14]. Another aspect to consider for graphene based materials is represented by their variable bonding arrangement with not well-defined stoichiometry [15]. Therefore, well characterized protein/carbon-based nanomolecule complexes are really sought for stimulating studies involving hybrid materials.

Since 2008, protein crystallography has been instrumental in understanding the nature of interactions between proteins and carbon-made nanomolecules and provided insights for chemical modifications of these materials [16,17,18]. Although structure determination of these complexes is not trivial considering the size and often the scarce solubility of these carbon-based nanomolecules, [16] use of nanomolecules sparked a strategy for crystallization of difficult biological macromolecules [19,20]. For instance, graphene wrapped protein crystals protect from dehydration and stabilize disordered surface solvent molecules, therefore improving crystal diffraction [21]. A number of fullerene derivatives were reported to inhibit important drug target enzymes as HIV-1 protease and acetylcholinesterase a key enzyme for nervous system activity [22,23,24]. A metal hydroxylated form of fullerene was designed as a potential anti-metastatic agent for pancreatic cancer [25,26]. In addition, a number of chemical approaches are available to functionalize fullerenes or nanotubes and alter their proteins binding ability [27,28,29,30,31,32,33].

Precise understanding of interactions between proteins/nucleic acids and carbon-based nanomolecules provide crucial insights to improve safety aspects concerning the use of these materials [33,34,35,36,37].

Beginning with the pioneering studies of Brian Matthews with lysozyme phage T4 mutants, aimed to understand the minimal requirements for protein folding stability and how it can be rescued by binding to nonpolar or very slightly polar ligands such as benzene molecule [38], the selection of a stable protein with high affinity for a given nano-particles became a very attractive topic [39,40]. Typical nanomolecules involve protein interfacing interactions in much larger numbers than smaller probes. 

The search for proteins as a binder of a given nanomolecule is not trivial because of many factors to balance between van der Waals and solvation interactions [17,41]. The first well-characterized synthetic protein aimed to solubilize a carbon nanotube through its coating consists of an amphiphilic α-helix containing hydrophobic and aromatic residues used to pack against the nanotube wall to improve interface affinity [42,43]. Aromatic amino acids are known to play a central role for protein tertiary structure stability and facilitate protein folding by reducing intramolecular hydrogen bonds around large aromatic residues [44,45,46]. Peptide sequences with high affinity for carbon nanomolecules show aromatic residues such as histidine or tryptophan residues and are characterized by flexible segments [47,48]. Similar considerations can be drawn for the affinity between nucleic acid and a nanotube [49]. 

The search for a protein as a good nanomolecule binder is reversed with respect to a search for a small ligand binder for which libraries are available [50]. Protein selection for a nanomolecule using high-throughput virtual screening or cheminformatics analysis are available [51,52].

Earlier crystallographic studies with cyclodextrins, a family of macro-cyclic oligosaccharides, and other studies fullerenes interacting with proteins inspired use of hollow shaped host-guest carbon-based nanomolecules that could potentially bind to proteins: cryptophanes, calixarenes, cucurbiturils, tweezers, etc. [53]. For these classes of organic molecules, besides aromatic residues hydrophilic and charged residues like arginine, lysine is important for protein–nanomolecule interactions [54,55,56]. 

In this review, we focus on the crystal structures of carbon-based nanomolecules interacting with proteins to highlight conserved structural aspects among the different types:

1. Fullerene and nanotubes;
2. Cryptophane and macrocages;
3. Supramolecular host-guest molecules: cyclodextrins, calixarenes, cucurbiturils, and molecular tweezers.

Searches for the above carbon-based nanomolecules were conducted in the Chemical Component Dictionary (CCD, e.g., ligands) and 3D structures of proteins in complex with these nanomolecules available in the Protein Data Bank (PDB; https://www.wwpdb.org) were identified from RCSB PDB website (https://www.rcsb.org) [57,58]. The 3D structures and stabilizing intra- and inter-molecular interactions were visualized for each example of a nanomolecule type. Primary literature and reviews were consulted to understand the functional implications of the interactions. For each type of nanomolecule, the chemical component identifiers, functional properties, and potential applications are described in the following sections and summarized in Table 1. The computed values of solvent-accessible surface area (Å^2^) for each of the nanomolecules in complex with a protein and the area of the uncomplexed form, as performed by using the server Ligand–Protein Contacts are reported in Table 1 [59]. In addition, the server provides the total atomic contacts between ligand and protein and lists putative hydrogen bonds.

## 2. All-Carbon Nanomolecules Interacting with Proteins

A widespread method used by researchers to promote protein affinity towards fullerenes (or nanotubes) is the use of covalently linked pyrenyl group anchored to the protein through surface lysines. Pyrenyl behaves as a molecular “glue”, able to stick to the nanotube wall via non-covalent π-stacking interactions [60].

**Figure 1:**
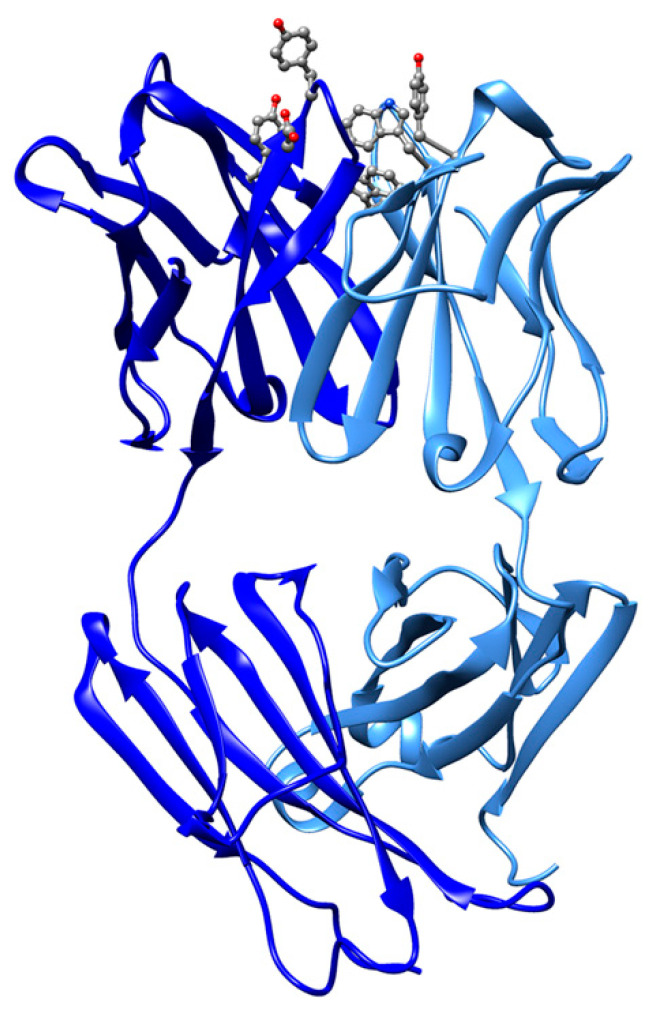
Ribbon drawing of mouse antifullerene antibody Fab-C_60_ (pdb entry 6H3H). The structure of Fab-C_60_ (complex of heavy (H) and light (L) chains) shows a fullerene binding pocket consisting of a canonical CDR region that contains various aromatic residues and an aspartate residue highlighted in balls-and-sticks (O red, N blue, C gray).

Immunization of mice with fullerene derivatives represents another method of producing in vivo IgG antibodies with high affinity towards fullerene (or nanotubes) [61,62]. With this approach, papain-cleaved Fab-IgG chains were obtained and purified and they showed high affinity for fullerene, measured in 22 nM [63]. The unbound Fab-IgG chains structure was solved by X-ray crystallography (pdb entry ID 1emt) [63]. Similarly, a recent antifullerene antibody Fab-C_60_ was obtained from mouse immunization and the structure of the complex of heavy (H) and light (L) chains solved by X-ray crystallography (Figure 1, pdb entry 6H3H) [64]. The structure of Fab-C_60_ shows a binding pocket consisting of a canonical CDR region that contains various aromatic residues (Tyr50 (H), Tyr101 (H), Tyr34 (L), Trp93 (L), and Trp98 (L)) and an aspartate residue (Asp100 (L)) [64]. The segment Asp100-Tyr101 solvent exposed the conformational disorder and is postulated to facilitate fullerene binding [64].

This approach was also used to select Fab chains with a high affinity towards a nanotube [65]. Another in vivo approach is the phage display technique that allows peptide selection from a library in presence of a nanotube used as target. During rounds of evolution while bacteria is infected, a peptide gene of higher binding affinity is isolated [47].

**Figure 2:**
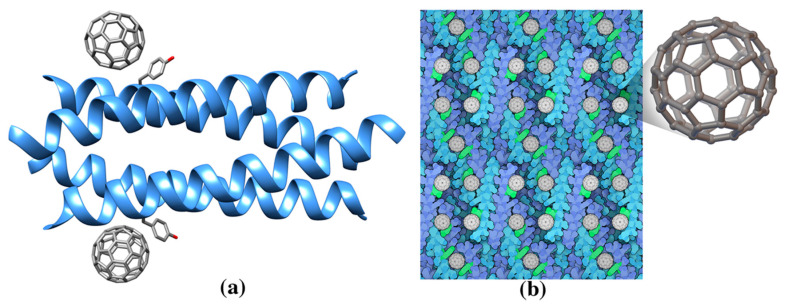
(**a**) Ribbon drawing of the de novo designed protein COP (C60-organizing peptide) in complex with fullerene (pdb entry 5et3). (**b**) COP protein in complex with fullerene forms a large superstructure. Each fullerene molecule is bound to two four helix bundles through the side chain of a Tyr residue (green). This figure is obtained from the Molecule of the Month column “Proteins and Nanoparticles” (pdb101.rcsb.org/motm/222). Inset: fullerene molecule from the crystal structure of COP-fullerene complex (gray spheres, cif code 60C).

Among methods to select proteins with good affinity for nanotubes de novo design offered an exemplary strategy. Researchers noticed that the geometry of an ideal alpha helix matches the honeycomb geometry of graphene. So, they positioned alanine amino acids along an alpha helix to match the center of the repeating hexagonal unit of the graphene sheet. Then, they engineered interactions based on a previously designed four-helix bundle in order to wrap helices around the nanotube. As expected, the designed peptide composed by the following thirty amino acids sequence —AEAESALEYAQQALEKAQLALQAARQALKA—binds to nanotubes and its structure solved by X-ray crystallography shows an Ala-rich surface in agreement with the designed peptide (named Hexcoil-Ala, pdb entry 3s0r) [66]. Serendipitously, the designed alpha helix, called COP (C_60_-organizing peptide), forms a crystalline complex also when mixed with buckminsterfullerene. The crystal structure of COP (Figure 2, Table 1, pdb entry ID 5et3) shows how the peptide, organized in a four-helix bundle motif (Figure 2), recognizes the fullerene with their tyrosine amino acids. Each nanomolecule is sandwiched by two four-helix bundles forming a large superstructure (Figure 2b) [67]. Astonishingly, when tested, fullerenes or COP proteins by themselves are not conductive, but the hybrid material with this 3D lattice does conduct electricity.

In summary, different strategies from in vivo selection or through de novo design are available to produce artificial proteins/peptides with high affinity to fullerenes and nanotubes. Fullerene does interact with Tyr and the methyl group of an Ala, or other aromatic residues properly placed in a protein sequence, resembling nanoparticle geometrical periodic features.

## 3. Carbon-Based Nanomolecules Interacting with Proteins

Typical carbon-based nanomolecules interacting with proteins are organic macrocycles widely used in supramolecular host–guest chemistry. Crown ethers, cyclodextrins, calixarenes, porphyrins, cryptophanes, molecular tweezers, cucurbiturils and organic foldamers resembling DNA are examples of host molecules for recognitions of guest counterparts, organic and metal ions, peptides and other organic molecules [68,69,70]. Considering the size and chemical properties of nanomolecules interacting with proteins, one major functional implication is the possible modification of the protein quaternary structure. Therefore, the use of these nanomolecules is potentially important to modulate many biochemical signals based on protein–protein interactions [71,72]. A recent perspective paper highlighted synthetic host molecules interacting with proteins and available structural data, and the modulation of protein function typical of supramolecular chemistry [73]. Crystal structures are particularly important in this field, because they are used as starting point for “retrostructural” analysis in order to improve further design of a specific nanomolecule interacting with a protein [74].

### 3.1. Molecular Cages

Pines and collaborators used cryptophanes and cyclodextrins, carbon-based macrocages, for xenon binding to develop protein binding materials for biosensing technique [75,76]. An isotope of xenon (129-Xe) is used as a contrasting agent for magnetic resonance imaging (MRI) in medical diagnostic testing, both as a gas to image airspaces in the lung and dissolved in body fluids to image the bloodstream and tissues [77].

**Figure 3:**
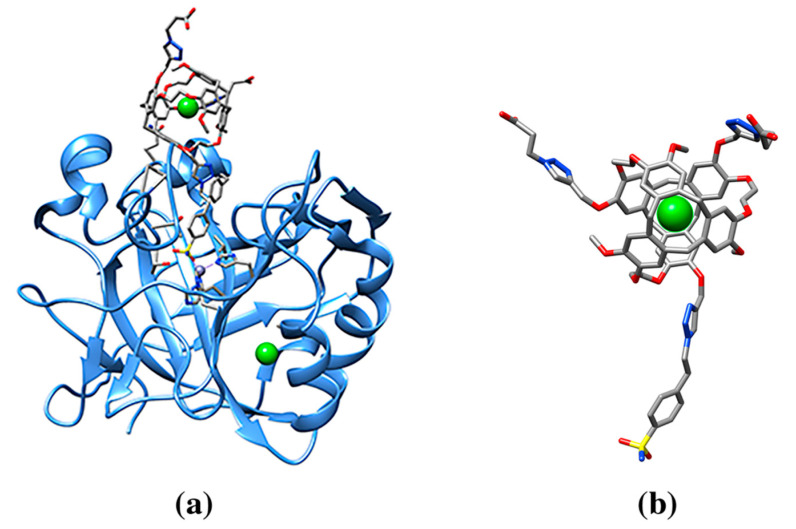
(**a**) Ribbon drawing of human carbonic anhydrase in complex with cryptophane-xenon (pdb entry 3cyu). The cryptophane-xenon is anchored with its benzenesulfonamide group to the zinc ion (purple) active site aromatic residues and an aspartate residue highlighted in balls-and-sticks (O red, N blue, C gray). (**b**) Cryptophane-xenon molecule (Xe green sphere, ligand cif code 0CR, or its enantiomeric molecule cif code 1CR).

Cryptophanes are molecular cages chemically made by linking two cyclotriveratrylene cups to form a hollow shell through -CH2-CH2- or other aliphatic and ether linkers. The overall shape and size of the cage allow for a dynamic entry and exit of small gas molecules [78,79]. A specific cryptophane was designed for binding to carbonic anhydrase, an enzyme that interconverts carbon dioxide and bicarbonate. The studied cryptophane bears two functions. On one hand, it is composed of a right sized cage for good affinity of a single xenon atom, and on the other hand, it is branched with a known benzenesulfonamide inhibitor to bind the zinc ion of carbonic anhydrase active site. This designed macrocage has a good affinity for the enzyme with a KD = 100 nM, measured by ITC. Xenon, when trapped to cryptophane, displays a distinctive MRI spectrum [16]. The structure of human carbonic anhydrase II in complex with cryptophane-xenon represented the largest ligand known in the Protein Data Bank by 2008 (ligand codes 1CR, 0CR). It displays bound xenon in the central core through non-covalent interactions, while cryptophane is anchored to the zinc ion through a benzenesulfonamide group (Figure 3, Table 1, pdb entry 3cyu). The main interactions between the macrocage and the enzyme are van der Walls interactions, and about a third of the total surface area available to solvent (Table 1) is buried in the active site of the enzyme. The quaternary structure of the enzyme is also affected as a consequence of significant crystallographic contacts occurring between symmetry-related macrocages buried within the enzyme [16]. In summary, macrocages can be used to target a specific enzyme in order to deliver noble gases for bioimaging applications, and they can function as promoter of large protein assemblies. An inhibitor or a ligand with a good protein affinity is combined with macrocage, which in turn is selective for specific gas molecules.

### 3.2. Calixarene Molecules

Calix[*n*]arenes are formed by three or more aromatic monomers (“-arene”) arranged in a cyclic structure that adopt an overall “calix” shape [80,81,82]. Calix[*n*]arenes upper and lower rims are functionalized with various chemical groups as hydroxyl groups, negative sulphonate groups, or positive amidinium groups (Table 1). These groups can improve solubility, shape and hosting properties [80]. Calix[*n*]arenes can bind metal ions, small ligands, biogenic polyamines, peptides, and proteinogenic charged residues such as lysine and arginine, and can form molecular capsules [83,84,85,86,87,88].

The first example of calixarene use for quaternary structure modulation is represented by a mutant (R337H) of tumor suppressor protein p53. This mutant promotes tumor growth because of the protein’s inability to form its natural tetrameric state that bind to the genome [89]. The calix[4]arene, functionalized with positive guanidiniomethyl groups at the upper rim and neutral hydrophobic loops at lower rim, rescues the functional activity of the protein restoring its quaternary structure [90]. Therefore, this study is exemplary for protein–protein interactions assisted by calixarene molecules, however, no experimental crystal structure is available for this complex.

**Figure 4:**
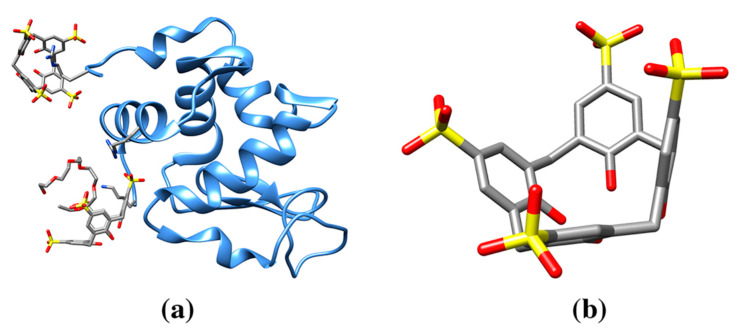
(**a**) Ribbon drawing of egg-white lysozyme in complex with sulfonatocalix[4]arene (sclx_4_) (pdb entry 4prq). The structure shows one calixarene molecule “cupping” an arginine side chain and therefore, camouflages the surface amino acid positive charge. The second calixarene molecule hosts a PEG molecule fragment (stick representation) from crystallization medium. (**b**) Sulfonatocalix[4]arene molecule (cif code T3Y, S yellow).

The first X-ray structure of a calix-protein crystal reported by Crowley and colleagues is the complex between a negatively charged calix[4]arene and cytochrome-c, an electron carrier protein with a surface containing a large number of positively charged residues [91] (pdb entry 3tyi, Table 1) [92]. The structure revealed the ability of the sulfonatocalix[4]arene (sclx_4_) molecule to explore and camouflage the lysine positive charges [92]. Similarly, the structure of the complex between egg-white lysozyme and sclx_4_ revealed the calix molecule bound to enzyme surface (pdb entry 4prq, Table 1, Figure 4) [93]. However, in this study, calixarene molecules behave in two different ways. One molecule binds and “camouflages” the charge of an arginine amino acid on the lysozyme surface, and the other hosts a PEG molecule from crystallization medium. These interactions allow calixarene to promote the assembly of lysozyme to form tetramers, which, in turn, further assemble into long repeating chains in the crystal.

The sclx_4_ molecule is also able to selectively recognize post translational modifications of lysine residues, as observed in the crystal structure of sclx_4_ bound to dimethyllysine residues of lysozyme (pdb entry 4n0j, Table 1) [94].

The binding property of calixarene to proteins was then used to explore the ability of calixarene-bioconjugates to promote non-covalent PEGylation, which can increase the half-life of therapeutic proteins. In this proof-of-concept exercise, mono-(pdb entry 6egy) or di-(pdb entry 6egz) PEGylated sulfonatocalix[4]arene are bound the cytochrome-c (Table 1) similar to the parent sclx_4_ [95].

In another example, protein recognition was explored to study the interaction of cytochrome-c with a series of sclx_4_ derivatives where one sulphonate group at the upper rim is replaced with a bromine (pdb entry 5lft, Table 1) or a phenyl group (pdb entry 5kpf, Table 1) [96]. Substituted calixarenes are bound to different lysine residues in function of specific chemical properties of the substituents: the -phenyl derivative packs against the protein through a hydrophobic cluster, while the -bromine substituted calix interacts with the carbonyl group of its bound lysine [96]. Therefore, calixarenes can be used as programmable molecules to control specific protein assemblies or to guide their binding towards a selected surface region either for design purpose or a probe to “hide” undesired genetic mutations.

**Figure 5:**
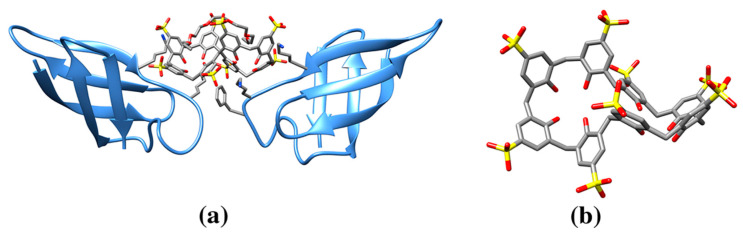
(**a**) Ribbon drawing of fungal protein PAF in complex with sulfonatocalix[8]arene (**sclx**_8_) (pdb entry 6haj). The structure shows a calixarene molecule sandwiched between two protein molecules with a conformation accommodating the nanomolecule binding through several charged and hydrophobic residues. A PEG-molecule is hosted in the cavity of **sclx**_8_ (**b**) Sulfonatocalix[8]arene molecule (cif code EVB, S yellow).

In the series of sulfonato-calix[*n*]arenes sclx_n_, the increasing number of arenes (letter *n*) increases the net charge of the scaffold and contemporarily increases the dimension and flexibility of the macro-ring. Calix[4]arenes are generally more pre-organized rigid cones with respect to calix[6]arenes, and even more with respect to calix[8]arenes. These negatively charged molecules function like “molecular glue” interfacing two or more proteins. The X-ray structures of cytochrome-c crystallized with the series sclx_n_ (*n* = 4, 6, 8) evidence an increased porosity of protein crystalline frameworks with an increasing calix[*n*]arene dimension. In particular, both sclx_6_ (pdb entry 6rgi) and sclx_8_ (pdb entry 6gd6, Table 1) induce highly porous assemblies of cytochrome-c (Table 1). While sclx_4_ shows a normal protein crystal packing (~45% solvent content), sclx_6_ yielded a honeycomb arrangement (~65% solvent content) [97] and sclx_8_ mediated a high-porosity framework (~85% solvent content) [98]. Owing to their ‘floppiness’, sclx_6_ and sclx_8_ can reshape to the protein surface and form large interfaces. Recently, it was shown that calix[8]arene conformation changes (Figure 5) are mediated by an effector, PEG-molecule (pdb entry 6haj, Table 1) or spermine (pdb entry 6rsl, Table 1), which in-turn modulates the porosity of cytc–sclx_8_ assemblies (~70% solvent content) [99].

**Figure 6:**
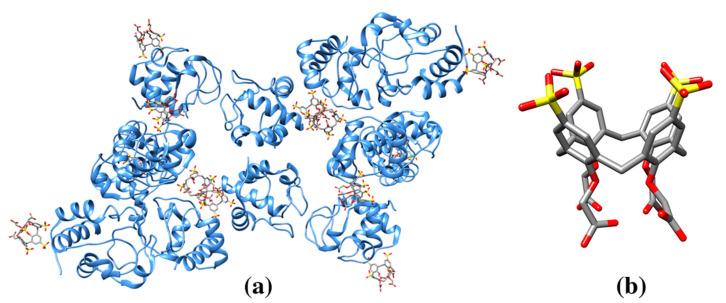
(**a**) Ribbon drawing representation and crystal packing of yeast cytochrome c in complex with octa-anionic calix [4] arene (**sclx4mc**, pdb entry 6haj). The negative charges of calixarene molecule are involved in electrostatic interactions with the positive charges of specific arginine and lysine residues from protein molecules crystallographically related. (**b**) Octa-anionic calix[4]arene molecule (cif code LVT).

However, the observed trend in protein crystalline open frameworks can be ascribed not only to the flexibility of the supramolecular mediator, but possibly also to its increasing net charge. The effect of the increase in these two properties was reported for few sclx_n_ examples. In order to probe the determinants that contribute to modulate the crystal architecture, it is necessary to uncouple flexibility and charge, using a more rigid and, at the same time, more negatively charged calixarene; or using a more flexible and less negatively charged calixarene. However, due to solubility problems, the latter example is less compatible with protein co-crystallization experiments. Therefore, the former strategy was recently adopted and the assembly-inducing behaviour of an octa-anionic calix[4]arene, sclx_4_mc was investigated [54]. This compound is a sclx_4_ derivative with four carboxylate functionalities at the lower rim. In particular, the presence of four chelating oxomethylcarboxylate (O-CH_2_-COO^−^) units at the lower rim confers the ability of these podand-like calixarenes to coordinate metal ions [100,101]. Metal complexation rigidifies the cone structure (prevents ‘‘breathing of the calix’’) [102] and enhances the binding of cationic guests in the calix[4]arene cavity such as the meso-tetrakis(4-*N*-methylpyridyl)porphyrin [103,104]. Two crystal structures of sclx_4_mc in complex with yeast (pdb entry 6suy), or horse heart cytochrome-c (pdb entry 6suv) were obtained [54]. The calixarene binds to a similar site on each protein but different assemblies were observed from crystal structure studies of these complexes: a honeycomb arrangement of yeast cythocrome-c (Figure 6) (~75% solvent content) and a tubular assembly of horse cythocrome-c (~55% solvent content) [54]. Interestingly, in the less porous structure, one carboxylate unit of calixarene coordinates an arsenic atom derived from the cacodylate buffer. The comparison of the buried surface areas (Table 1) for the complexed ligand in the two structures shows that the extra arsenic atom causes a tighter crystal packing and a major enclosure by protein residues. The comparison with crystal structures obtained with the series sclx_n_ suggested that the ligand charge is a crucial contributing factor to porous architectures.

The property of sclxn (with *n* = 4, 6, and 8) molecules to facilitate protein crystallization was further investigated with a small antifungal protein, PAF. The role of calixarene-mediated assembly of this basic protein was confirmed in the PAF-sclx4 co-crystal (pdb entry 6ha4), PAF-sclx6 co-crystal (pdb entry 6hah) and PAF-sclx8 co-crystal (pdb entry 6haj) [105]. One of the PAF complex structures is shown in Figure 5.

Other water soluble calixarene variants were used for the complexation of cytochrome c: the p-methylphosphonatocalix[4]arene (pdb entry 5ncv) [106] and the phosphonato-calix[6]arene (pdb entry 5lyc) [107]. In summary, a variety of calixarenes derivatives were explored to understand the effects on protein binding. Smaller rigid calixarenes interact and explore long positive charges of surface Arg or Lys residues and can change protein surface electrostatic. Larger calixarenes change their skeleton conformation to bind, adapt to protein surface. Finally, calixarenes with multi charges cause different protein aggregations.

### 3.3. Cyclodextrins

Cyclodextrins are cyclic oligosaccharides formed by five or more glucose monomers linked by α-1,4 glycosidic bonds arranged in a cyclic structure. Cyclodextrins have a variety of applications for food and pharmacological industries. These molecules are biochemically produced from starch enzymatic digestion of α/β TIM-barrel fold hydrolytic enzymes. The best known cyclodextrins contain a number of glucose monomers ranging from six to eight glucose units, known as α-cyclodextrin (6 glucose units); β-cyclodextrin (7 glucose units) and γ-cyclodextrin (8 glucose units).

The cyclic repeat of glucose units causes a characteristic regular conformational shape of the ring. A search in the Protein Data Bank retrieves a number of hydrolytic enzymes crystal structures in complex to α- and β-cyclodextrins (ligand codes ACX and BCD, respectively) where the bound oligosaccharides shows a regular conformation described by their six- or sevenfold axis. For instance, the crystal structure of the amylase soybean β-amylase in complex with α-cyclodextrin revealed a leucine side chain (Leu 379) hosted in the hydrophobic cavity of the cyclic oligosaccharides (pdb entry 1btc) [53]. Notably, while all six glucose units of the α-cyclodextrin lie essentially in the plane of the oligosaccharide, it adopts a toroidal shape that resembles calixarene with a hydrophobic cavity.

The thermostable alpha-amylase enzyme in complex with α-cyclodextrin revealed a methionine side chain hosted in the cyclic sugar cavity and outstanding interactions with two Trp residues (Table 1, pdb entry 3bcd) [108]. Similarly, the crystal structure of cytochrome P450 in complex with vitamin D2 and β-cyclodextrin revealed a phenylalanine side chain from a surface loop (Phe 214) hosted within the oligosaccharide cavity (Table 1, pdb entry 3czh).

**Figure 7:**
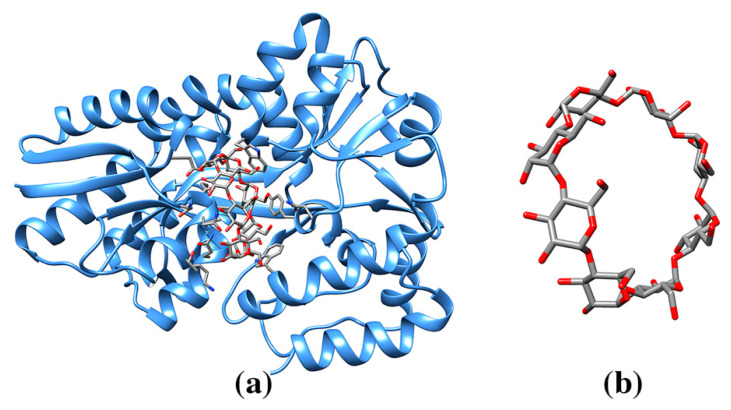
(**a**) Ribbon drawing of maltodextrin binding protein MalE1 in complex with γ-cyclodextrin (pdb entry 5mka). Cyclodextrin molecule hosts in its large cavity side chains of an asparagine and an alanine residue and in turn interact with the side chains of several aromatic residues. (**b**) γ-cyclodextrin molecule (cif code RCD).

Because of these characteristic conformational properties cyclodextrins present relevant host-guest properties and are often used to build supramolecular architectures. However, there are exceptions to the symmetrical conformation of an oligosaccharide. The conformation of the macrocycle could deviate from a regular arrangement of the sugar moieties especially for larger oligosaccharides, or to accommodate a specific hydrolytic mechanism. For instance, the crystal structures of maltodextrin binding protein MalE1 or cyclomaltodextrinase bound to γ-cyclodextrin show a bending of the oligosaccharide. In addition, different pairs of residues are hosted in the cavity of the γ-cyclodextrin (ligand codes RCD) when the crystal structures of the two enzymes are compared (Asn/Ala; pdb entry 5mka, Figure 7; and Arg/Glu, pdb entry 3edk) and a variety of aromatic protein residues interact with the external surface of the cyclic sugar (Table 1) [109,110,111]. In summary, cyclic polysaccharides of different sizes form an inner cavity that adapts to interactions with hydrophobic residues Leu or Phe (smaller rings) and aromatic residues.

### 3.4. Cucurbituril Molecules

Cucurbituril molecules are formed by the condensation of five or more monomeric units of glycoluril and formaldehyde arranged symmetrically in ringed structures with an overall characteristic pumpkin shape [112,113,114]. For instance, the hexameric macrocyclic compound, cucurbit[6]uril has a cavity with ~5.8 Å diameter and a portal (narrower entrance) of 3.9 Å diameter [115]. Cucurbiturils are used as joining bead molecules for building supramolecular architectures [113]. Cucurbiturils chemical structure form spatial dipoles that favor binding of a variety of pyridinium-based molecules, metal ions, cationic organic molecules, gas molecules [115].

There are several examples illustrating interactions between cucurbiturils and proteins. Cucurbit[7]uril, composed of seven monomer units, was used as a synthetic receptor for human insulin. The crystal structure of human insulin in complex with cucurbit[7]uril revealed the *N*-terminal phenylalanine residue of the hormone hosted in the core of cucurbit and the nitrogen amino acid interacting with the oxygen atom group of the host molecule (Table 1, pdb entry 3q6e) [116]. Cucurbiturils have the function to regulate protein interactions, as demonstrated with cucurbit [8]uril, which is able to recognize an epitope of a signaling tetratricopeptide repeat (TPR) binding protein 14-3-3 (pdb entry 5n10, Table 1) [73,117].

**Figure 8:**
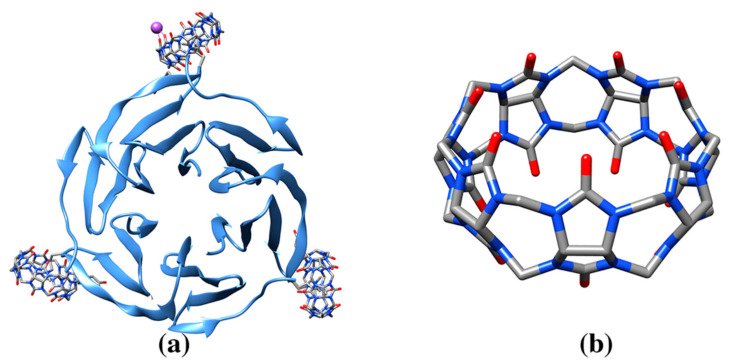
(**a**) Ribbon drawing of fucose binding lectin protein in complex with cucurbit[7]uril (pdb entry 6f7w). Cucurbit[7]uril molecules show selective binding towards post translational modifications of a surface lysine residues. One of the bound cucurbit[7]uril molecules interact with a sodium ion (Na purple). (**b**) Cucurbit[7]uril molecule (cif code QQ7).

**Figure 9:**
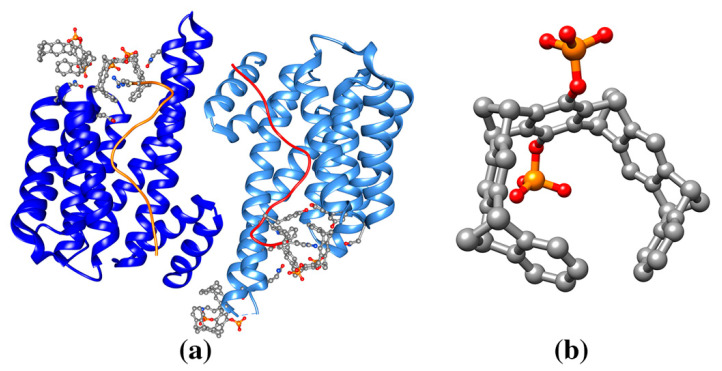
(**a**) Ribbon drawing of binding protein 14-3-3 protein zeta/delta in complex with phosphatase peptide (orange string) and molecular tweezer **CLR01** (pdb entry 5m37). The cavity of **CLR01** molecules host a side chain of an arginine or a lysine residue. (**b**) **CLR01** molecule (cif code 9SZ).

Cucurbit[7]uril is able to regulate the protein quaternary structure of lectin binding protein. The structure of lectin binding protein in complex with cucurbit[7]uril (Figure 8, Table 1, pdb entry 6f7w) reveals the selective binding of the nanomolecule for post translational modifications of lysine residues, similar to those observed for the structure of sclx_4_, with the side chain of the surface residue hosted within the capsule inner cavity (Figure 9) [118]. The crystal packing reveals formation of an ordered cucurbiturils cluster that promote assembly of the protein. This study suggests a use of cucurbiturils as a strategy to engineer complex and specific protein architectures [118]. In summary, the rigid and symmetrical skeleton of cucurbiturils is well suited to interact with phenylalanine and methylated side chain lysine and form clusters to direct protein assembly.

### 3.5. Molecular Tweezers

Among the nanomolecules able to host protein amino acids side chains, C-shaped “molecular tweezers” revealed interesting properties for promoting protein–protein interactions. The best-known tweezer, CLR01, is composed by alternating norbornadiene and benzene chemical groups forming a ring. A phosphate anion group is bound to the upper and lower rim of the CLR01 similar to the charged groups on calixarene rims (Table 1) [119]. The phosphate groups on CLR01 carbon skeleton improve water-solubility and binding to positively charged residues of lysine and arginine [120]. The molecule CLR01, with promising properties for developing Alzheimer’s disease therapy, has the ability to cause disruption of hydrophobic and electrostatic interactions proving its inhibition of nucleation and oligomerization of amyloidogenic proteins [121]. For similar reasons, CLR01 functioned as an inhibitor of superoxide dismutase (SOD1) aggregation. This dimeric copper-zinc enzyme, responsible for clearing our cells from toxic and reactive radicals (O_2_^−^), contains eleven surface lysine residues and, therefore, it is a good target for tweezer molecules [122,123]. However, no X-ray structures revealing details of interactions for these examples are available as of this writing.

Crystal structure determination of the complex between a signaling tetratricopeptide repeat (TPR) binding protein 14-3-3 and CLR01 revealed the mechanism that modulates protein–protein interactions [73]. Binding of 14-3-3 proteins by inactive kinase (e.g., Raf kinase-1), is linked to a number of malignancies and developmental syndromes Noonan and LEOPARD, and therefore, represents a target for drug discovery [124,125]. The comparison between the crystal structure of the binary complex 14-3-3 protein zeta/delta and peptide binding region of Raf kinase-1 (pdb entry 3nkx) and the complex between 14-3-3 protein zeta/delta and CLR01 (pdb entry 5oeh) reveals the potential interface for the tweezer inhibition mechanism [73,126,127]. CLR01 hosts a single surface-exposed lysine near to the binding of a symmetrically equivalent 14-3-3 protein zeta/delta [73]. Recent structure determination of 14-3-3 protein zeta/delta in complex with peptide binding region of M-phase inducer phosphatase 3 (another binding partner of 14-3-3 proteins) soaked with CLR01 (pdb entry 5m37, Table 1) (Figure 9). Therefore, CLR01 can tune protein–protein binding interactions beyond the simple inhibition mechanism. The binding of the CLR01 in this ternary complex (pdb entry 5m37) reveals a C-terminal peptide arginine residue being hosted in the inner core of CLR01 and stabilized by van der Waals interactions with neighboring residues and an electrostatic interaction with one of phosphate group [128]. Furthermore, this structure reveals the molecular basis for higher binding affinity of the peptide measured in presence of CLR01 molecule. Intrinsic disorder is a key feature of partners that bind 14-3-3 proteins and, therefore, tweezer can provide useful insight in how to stabilize these interactions [128,129]. In summary, CLR01 can be used to tune protein–protein interactions by affecting the binding affinity of specific proteins/peptides, or the changing the dynamic flexibility of intrinsically disordered proteins [125,130]. A rigid C-shaped skeleton combined with a negatively charged phosphate group is well suited to interact and explore long positive charges of surface Arg or Lys residues.

## 4. Conclusions and Outlook

For each carbon-based nanoparticle discussed in this review, we indicated a brief summary. A recent nanomaterial database resource (PubVINAS) archives a total of 705 unique nanomaterials corresponding to twelve materials types [25,131]. At the time of this writing (July 2020), eighty of these nanomaterials are represented by carbon nanotubes, forty-eight by C_60_ fullerene derivatives, and twenty by carbon nanoparticles. Carbon-based nanomolecules research is rapidly growing due to potential applications ranging across biological, medical, and material sciences [132,133]. Applications involving multifunctional cyclodextrins, used for molecules delivery, received a widespread interest and are already in use for clinical purposes [134]. The gathering of carbon-based nanomolecules with biological samples has the potential for trending areas of medical chemistry including protein–protein interactions and conformational flexibility of disordered proteins for which metal based nanomolecules were explored [135].

In order to improve the property of carbon-based nanomolecules and address their safety for medical use, it is crucial to have a clear understanding of their interactions with a target protein [18,132]. X-ray crystallography proved instrumental to understand the key interactions of proteins and carbon-based nanomolecules and inspired many of the studies we reviewed. Although these interactions are similar to those involving typical small molecules, the presence of a larger number of aromatic groups in carbon-based nanomolecules implies an important role of π-interactions (see Table 1). Despite the binding of large size carbon-based nanomolecules, these ligands often have a negligible effect on protein overall shape [16]. Carbon-based nanomolecules have the propensity to cluster because of their significant radii and rigid skeletons with resulting effect on protein quaternary structure [93]. Carbon-based nanomolecules coupled with charged or other chemical groups could change protein electrostatic surface [90]. Carbon-based nanomolecules can be used as framework to tune crystalline porosity by simple use of common buffer molecule as an additive.

Therefore, new chemical modifications of carbon-based nanomolecules have potential as creative ways to address specific questions involving targeted proteins. Lessons learned from structural studies examined here are exemplary for the future use of carbon-based nanomolecules to stoichiometrically combine a number of protein entities to build functional hybrid materials [67,136,137].

## Figures and Tables

**Table 1:** Crystal structures of protein binding to nanomolecules or protein-nanomolecule complexes discussed in this review and retrieved from the Protein Data Bank. Nanomolecule buried surface area upon complex formation (Å^2^), surface areas of individual nanomolecule (uncomplexed, Å^2^), and protein and/or nanomolecule function are indicated. Ligand chemical structures and other ligand annotations can be retrieved using the indicated nanomolecule cif code from the following link: https://www.rcsb.org.

Carbon-Based Nano-Molecule Name	Cif Code of Bound Nanomolecule	Proteins Interacting with Nanomolecules, (Identifiers of PDB Entry and Number of Bound Nanomolecules)	Main Interacting Protein Residues. Identifiers of PDB Entry, Nanomolecule, Protein Chain(s), Residue(s) or Atom(s), and Total Atomic Contacts	Buried Surface (Å^2^) Versus Nanomolecule Surface Area (Å^2^)	Protein and Nanomolecule Functions
Fullerene	-	Fab antibody fragment on an antifullerene antibody (1EMT: unbound)	1EMT: CDR region contains aromatic residues: Y36(L), W47(H), Y91(L), F96(L); residues N35(L), Q89(L)	*n*/a	Antibody binding fullerene.
Fullerene	-	Fab antibody FabC 60 on an antifullerene antibody (6H3H: unbound)	6H3H: CDR region contains aromatic residues: Y50(H), Y101(H), Y34(L), W93(L), W98(L); residue D100 (L)	*n*/a	Antibody binding fullerene.
Fullerene, buckminsterfullerene	60C	Fullerene organizing protein (C60SOL-COP-3) (5ET3: 1, 5HKN: 1, 5HKR: 1)	5ET3: 101 A Y9/A6/E2/S5 5HKN: 101 B Y9/A6/E2/S5 5HKR: 101 B Y9/A6/E2/S5	388/547 381/542 316/545	Designed peptide binding fullerene. Crystal of COP protein in complex with fullerene does conduct electricity
Single wall nanotube (SWNT)	-	De novo designed helical assembly (Hexcoil-Ala) (3S0R: unbound)	3S0R: Ala-rich HexCoil-Ala	*n*/a	Designed virus-like protein assembling on a carbon nanotube
^129^Xe-Cryptophane biosensor (racemic mixture)	0CR, 1CR	Human Carbonic Anhydrase II (3CYU: 1)	3CYU: 263A Q136/ZN/XE (158)	993/1515	Enzyme lyase. Nanomolecule functions as an inhibitor of the enzyme and a xenon biosensor
Sulfonatocalix[4]arene (sclx_4_)	T3Y	Cytochrome c (3TYI: 3, 4YE1: 3, 4N0K: 3)	3TYI: 105A K89 (83), 105B K4 (63), 106B K22 (60)	472/815, 610/826, 622/808	Electron carrier protein. Calixarene “camouflages” protein surface charges or promote quaternary structure formation.
			4YE1: 202A K89 (79), 202B K4 (71), 203B K89/K5 (48)	486/814, 589/840, 601/823	
			4N0K: 202A K89/K87 (84), 202B K4 (64), 203B K22 (60)	482/815, 627/830, 625/814	
		Lysozyme C (4PRQ: 5, 4PRU: 4, 4N0J: 4)	4PRQ: 201A R128 (67), 202A P6G (163), 201B R128 (68), 202B P6G (171), 203B PG4 (112)	598/826, 174/830, 593/825, 167/833, 426/835	Antibacterial protein
			4PRU: 201A MeK116 (70), 202A R14 (103), 201B MeK116/R112 (127), 202B R14 (97)	533/794, 459/815, 316/800, 483/805	
			4N0J: 201A MeK116 (73), 202A R14 (85), 201B MeK116/R112 (102), 202B R14 (86)	534/789, 506/812, 426/797, 488/800	
		PAF (6HA4: 1)	6HA4: 202A K30 (70)	545/824	Antifungal protein
		Lectin protein (6GL5: 6)	6GL5: 101A MeS1/T3Y (97), 101B MeS1 (73), 102B PEG (92), 101C MeK83 (57), 102C MeS1//T3Y (82), 103C S57 (80)	481/806, 558/813, 524/828, 642/812, 502/807, 487/831	Fucose-binding lectin protein.Calixarene is recognized by methylated serine
PEGylated sulfonatocalix[4]arene	B4T	Cytochrome c (6EGY: 4)	6EGY: 203A Y97/K100/K4 (101), 201B K73 (47), 203B B4T/K86 (135), 204B R13/B4T (63)	430/824, 636/840, 368/922, 133/419	Calixarene derivative as a probe to increase protein half-life.
Di-PEGylated sulfonatocalix[4]arene	B4X	Cytochrome c (6EGZ: 4)	6EGZ: 202A R13/B4X (51), 202B K27/B4X (78), 203 B K86/K87/B4X (137), 203B K72/B4X (73)	60/275, 123/463, 377/901, 655/889	
Bromo-trisulfonatocalix[4]arene	6VB	Cytochrome c (5LFT: 3)	5LFT: 204A K86/K87 (58), 205A 6VB/K89 (91), 206A 6VB (79)	559/776, 425/777, 501/776	Calixarene used as programmable molecule to control specific protein assemblies, or as a probe for “hiding” specific genetic mutations.
Phenyl-trisulfonatocalix[4]arene	6VJ	Cytochrome c (5KPF: 1)	5KPF: 202B K4/K5/(71)	621/854	
*p*-methylphosphonatocalix[4]arene	8TE	Cytochrome c (5NCV: 3)	5NCV: 202A K54/D50 (65), 203A K87 (74), 202B K86 (74)	682/941, 687/942, 651/932	Water soluble calixarene is used to study the effect on protein quaternary structure.
Sulfonatocalix[6]arene	FWQ	Cytochrome c (6RGI: 1)	6RGI: 202A R13/K73 (133)	668/1183	Increased calixarene size to study effect on protein crystallinity. Nanomolecule shows irregular conformation.
		PAF, Antifungal protein (6HAH: 1)	6HAH: K30/PEG (161)	690/1184	
Phosphonato-calix[6]arene	7AZ	Cytochrome c (5LYC: 2)	5LYC: 202A K4/K100 (67), 202B K4/K11/K100 (95)	900/1205, 835/1207	Water soluble calixarene is used to study the effect on protein quaternary structure.
*p*-sulfonatocalix[8]arene	EVB	Cytochrome c (6GD6: 1, 6GD7: 3, 6GD8: 3, 6GD9: 3, 6RSI: 3, 6RSL: 6, 6RSK: 6, 6RSJ: 6, 6GDA: 3)	6GD6: K4/K100/A8 (147)	1086/1538	Larger, eight membered ring calixarene and effect on protein crystallinity. Effect of a mediator molecule (e.g., spermine) between protein and calixarene.
			6RSI: 202A K27/K11/Q16/L15 (120), 203A GOL/K100 (165), 204A K73/K86 (159)	1158/1626, 928/1492, 1038/1665	
			6RSL: 202A K27/K11/Q16/L15 (131), 203A SPM/K100/K4 (190), 204A SPM/EVB/K86/K87 (223), 202B K27/K11/Q16 (125), 203B SPM/K100/K4(205), 204B SPM/EVB/K73 (223)	1163/1625, 819/1505, 753/1561, 1154/1627, 805/1510, 693/1559	
		PAF, Antifungal protein (6HAJ: 1)	6HAJ: PEG/K27/K30/F31(256)	720/1510	
Octaanionic calix[4]arene	LVT	Yeast cytochrome c (6SUY: 2)	6SUY: 202A R13/K92/K87 (83), 202B K87/K89/E88/D90 (59)	664/942, 783/954	Octa-anionic charged calixarene and effect on protein quaternary structure. Larger assembly is formed
	LVQ	Horse cytochrome c (6SUV: 8)	6SUV: 203A K86/K87/K88/T89/E90 (175), 203B K86/K87/K88/T89/E90 (161), 203C K86/K87/K88/T89/E90 (122), 203D K86/K87/K88/T89/E90 (120), 203E K86/K87/K88/T89/E90 (181), 203F K86/K87/K88/T89/E90 (172), 203G K86/K87/K88/T89/E90 (123), 203H K86/K87/K88/T89/E90 (123)	346/1031, 346/1009, 551/1017, 558/1028, 337/1001, 334/1021, 550/999, 534,984	
α-cyclodextrin	ACX	Thermostable alpha-amylase (3BCD: 1)	3BCD: 901A M176/W260/W287 (88)	649/991	Hydrolytic enzyme for starch degradation
β-cyclodextrin	BCD	Cytochrome P450 2R1 (3CZH: 1)	3CZH: 603A F240/P239 (120)	724/1119	Oxidoreductase
γ-cyclodextrin	RCD	Maltodextrin binding protein MalE1 (5MKA: 1)	5MKA: 401A A58/N59/N46/W234/Y164/W(353) (186)	560/1281	Protein belongs to the ABC transporter complex involved in maltose/maltodextrin import. Protein binds maltose and maltodextrins. Protein functions to linearize cyclomaltodextrin.
			3EDK: 700A R464/D466/D418/W342/ D311/F274/Y178 (180)	513/1263	
		Cyclomaltodextrinase (3EDK: 2)	700B R464/D466/D418/W342/D311/F274/ Y178 (196)	485/1273	
Molecular tweezer, CLR01	9SZ	14-3-3 adapter protein (5OEH:1, 5OEG:1, 5M36:3, 5M37:4)	5OEH: 301 A K214/Y213 (74)	579/832	Dimeric binding protein. Molecular tweezer is used as an inhibitor or a binding modulator.
			5M37: 301A N183/K138/9SZ301D (56), 301B K74/M78/E73 (121), 301C R208/N183 (138), 301D R208/9SZ301A (153)	640/837, 504/830, 363/833, 277/831	
Cucurbit[7]uril	QQ7	Human insulin (3Q6E: 1) Lectin binding protein (6F7W: 3, 6F7X: 2, 6SU0: 12)	3Q6E: F1/V2 (64)	827/1031	Hormone protein. Cucurbituril molecules are used as probe for engineering arrays of protein assembly. Methylated lysine residue is recognized
			6F7W: 101AMeK34 (71), 101B MeK34 (75), 101C MeK34 (74)	744/998, 712/993, 748/998	
			6F7X: 101AMeK34 (78), 103B MeK(34) (64)	749/996, 760/992	
			6SU0: 202A MeK34/Y37 (79), 102B MeK34/Y37 (83), 204C MeK34/Y37 (91), 201I MeK79B/Y82B (80), 201J MeK79C/W81C/Y82C (80), 201K MeK79A/W81A/Y82A (88), 201M MeK79D/W81D/Y82D (80), 202M MeK34F/Y37F (74), 201N MeK34D/Y37D (87), 202N MeK79E/W81E/Y82E (82), 201O MeK34E/Y37E (82), 202O MeK79F/Y82F (86)	742/1005, 754/1011, 694/1007, 749/1007, 717/1004, 717/1002, 716/991, 738/992, 712/999, 740/1001, 736/988, 708/992	
Cucurbit[8]uril	C8L	14-3-3 adapter protein (5N10: 1)	5N10: 601C: F581C/F581D (130)	643/1165	Dimeric binding protein. Larger size cucurbituril is used to study effect on protein assembly
